# Analysis of growth rate, haematologic, and biochemical parameters of Oncopigs

**DOI:** 10.1080/23144599.2025.2502711

**Published:** 2025-06-02

**Authors:** Lobna Elkhadragy, Caitlyn C. Castillo, Rena Li, Courtni R. Bolt, Laurie Rund, Faith Thomas, Rachel Lane, Ron C. Gaba, Lawrence B. Schook, Kyle M. Schachtschneider

**Affiliations:** a Department of Radiology, University of Illinois at Chicago, Chicago, IL, USA; b Department of Animal Sciences, University of Illinois at Urbana-Champaign, Urbana, IL, USA; c Center for Clinical and Translational Science, University of Illinois at Chicago, Chicago, IL, USA; d Department of Pathology, University of Illinois at Chicago, Chicago, IL, USA

**Keywords:** Oncopig, transgenic pig, large animal model, cancer model, haematologic and biochemical parameters, growth chart

## Abstract

Pigs are widely used as large animal models in biomedical research due to their physiological and anatomical similarity to humans. The Oncopig, a genetically engineered pig model harbouring Cre recombinase-inducible *KRAS*^*G12D*^ and *TP53*^*R167H*^ transgenes, serves as a valuable model for cancer research. This study describes the generation of Oncopig breeding herds and provides a characterization of their growth rate, body size, and physiological parameters, including haematologic, biochemical, and coagulation profiles. Body weight and size were measured in male and female Oncopigs, and blood samples were collected at multiple time points from birth to one year of age. A total of 13 haematologic, 18 biochemical, and 3 coagulation parameters were analysed. While male and female Oncopigs had a comparable growth rate within the first 6 months of age, male Oncopigs exhibited a significantly higher growth rate between 6 and 12 months of age and a higher body weight at 12 months. The mean body weight at 6 months of age was 52.3 kg for male Oncopigs and 48.0 kg for female Oncopigs, while at 11 months of age it was 96.4 kg for male Oncopigs and 71.7 kg for female Oncopigs. Haematologic, biochemical, and coagulation parameters were analysed for Oncopigs under 6 months of age, over 6 months, and collectively for Oncopigs within a year of age. By providing comprehensive data on growth, haematologic, and serum biochemical parameters, this study provides a critical resource for researchers utilizing Oncopigs as large animal models for cancer research and other translational studies.

## 1. Introduction

Pigs are one of the most frequently used animal models in biomedical research, translational studies, and surgical training [1–3]. This widespread use is primarily attributed to their resemblance to humans in physiology, metabolism, size, body weight, blood volume, capacity for clinical monitoring, lifespan, biochemistry, and pathogen response [1–3]. Monitoring the health status of animal models and detecting abnormalities is crucial for accurate disease monitoring and diagnosis. This can be accomplished with the guidance of established growth curves and clinical laboratory results, helping with assessment of animal health and diagnostic interpretation.

The Oncopig Cancer Model, or the Oncopig, is a transgenic pig model that recapitulates human cancer by developing site- and cell-specific tumours [4]. This inducible porcine model harbours a transgene that expresses *KRAS*^G12D^ and *TP53*^R167H^, selected due to the frequent occurrence of these mutations in human cancers. The *RAS* gene is mutated in approximately 19% of human cancers, with the *KRAS*^G12D^ isoform being the most common mutation yielding a constitutively active, oncogenic protein [5,6]. Similarly, the tumour suppressor gene *TP53* is mutated in about one-third of human cancers. The R167H mutation corresponds to the human R175H mutation, a commonly found dominant negative *TP53* mutation associated with tumorigenesis [7–9]. To develop the Oncopig transgene construct, porcine KRAS and TP53 cDNAs were cloned and mutated through site-directed mutagenesis [4]. The *KRAS*^G12D^ and *TP53*^R167H^ cDNAs were then introduced into a Cre-inducible vector, yielding an expression construct containing the CAG promoter, followed by Lox-flanked STOP sequence, KRAS^G12D^, an internal ribosome entry site (IRES) sequence for bicistronic expression, TP53^R167H^, and a poly A sequence. This design enables the co-expression of KRAS^G12D^ and TP53^R167H^ in any Oncopig cell upon transient exposure to Cre recombinase, which can be achieved by a viral vector [4].

Previous studies have established growth charts and reported haematologic, biochemical, and coagulation profiles for several pig breeds, including domestic and miniature pigs [10–14]. However, these fundamental data are currently unavailable for Oncopigs. As Oncopigs are developed by crossing a minipig transgenic sire harbouring the Cre recombinase inducible transgene encoding KRAS^G12D^ and TP53^R167H^ with a domestic breed dam, it is essential to characterize the growth rate, haematologic, and biochemical profiles of these crossbred Oncopigs.

The objectives of this study are twofold: 1) to describe the generation of Oncopig breeding herds, and 2) to analyse growth rates, body size, and physiological parameters in healthy Oncopigs. This study analyzes the growth rates as well as 13 haematologic, 18 serum biochemical, and 3 coagulation parameters in male and female Oncopigs between birth and one year of age. These results will provide researchers with essential information contributing to the refinement of research studies that utilize Oncopigs as a cancer model.

## 2. Materials and methods

### 2.1. Animals

This study was approved by The University of Illinois Institutional Animal Care and Use Committee (IACUC). The study included six female Oncopigs and four male Oncopigs monitored from birth to 12 months of age. Oncopigs were provided with water ad libitum and age-appropriate calorie-restricted diet (Supplementary Tables S1 and S2). The pigs were routinely examined by the University of Illinois at Urbana-Champaign (UIUC) veterinarians. Oncopigs were vaccinated for Porcine Parvovirus, Erysipelas, Leptospirosis, *E.* *coli*, and *C.* *perfringens* Type C in addition to being tested annually for *Mycoplasma hyopneumoniae*, Porcine Reproductive and Respiratory Syndrome virus (PPRSV), and Swine Influenza Virus.

### 2.2. Body weight and size measurement

Oncopig body weight (kg) was measured at several time points between birth to nearly 12 months of age (Supplementary Table S3). Oncopig body length from snout to tail (cm) and maximal abdominal or hip circumference (cm) were measured at different time points between 1 and 11.4 months of age (Supplementary Table S4). To compare male and female body weight and size values, linear mixed-effects models were fit for each outcome. Models included random subject intercepts and slopes and fixed effects for quadratic or cubic time, sex, and sex-by-time interaction, with the best-fitting structure for each outcome selected through likelihood ratio tests and Akaike Information Criterion (AIC) values. Model-based means and 95% confidence intervals are reported in the manuscript.

### 2.3. Blood collection

Blood was collected from the jugular vein of awake, restrained Oncopigs in the morning following a 12 hour fast. The blood was drawn into three vacutainers as follows. For haematology analysis, blood was drawn into a vacutainer containing ethylenediamine tetraacetic acid (EDTA) anticoagulant and immediately delivered to the diagnostic lab. For biochemical analysis, blood was drawn into a vacutainer with no additives and allowed to clot for 30 min before being centrifuged at 3,000 rpm for 10 minutes to separate the serum from the clot. The serum was then pipetted out and promptly submitted to the diagnostic laboratory. For coagulation parameter analysis, blood was drawn into a vacutainer containing Acid Citrate Dextrose (ACD) anticoagulant and placed on a rocker for 10 minutes, before being delivered to the diagnostic laboratory.

### 2.4. Blood analysis

Blood was used for analysis of 13 haematologic, 18 serum biochemical, and 3 coagulation parameters at the Veterinary Diagnostic Laboratory at the UIUC. All analyses were performed by the diagnostic laboratory on the same day of blood collection, immediately after receiving the samples, using Cell Dyn 3700 and Beckman Coulter AU680. The first blood collection occurred between 1 and 2.5 months of age, with subsequent collections performed once or twice per month thereafter (Supplementary Table S5).

### 2.5. Analysis of haematologic, biochemical, and coagulation parameters

Each outcome was graphically evaluated for normality and asymmetric distributions were transformed by the Box-Cox procedure. Outliers were detected by the Tukey fence method and values more than 1.5 interquartile range (IQR) below the first quartiles (Q1) or above the third quartile (Q3) of the transformed distribution were removed. After removal of outliers, values were characterized both nonparametrically and parametrically [15]. Descriptive statistics were calculated, including count, minimum, first quartile, median, third quartile, and maximum. Linear mixed-effects models were fit to each transformed outcome, with the best-fitting random effects structure (intercept-only or intercept and slope) and fixed effects (linear, quadratic, or cubic time terms, with and without adjustment for sex) selected through likelihood ratio tests and AIC values. Residuals were evaluated for approximate normality and homoskedasticity through histograms, scatter plots, and QQ plots. To capture the expected range of future observations, model-based predicted values were simulated through a semi-parametric bootstrapping procedure with 1,000 replications; 95% prediction intervals were estimated with 90% confidence intervals (CI) around each endpoint. Estimates and interval endpoints were then back-transformed to the original scale using inverse Box-Cox transformations.

## 3. Results

### 3.1. Oncopig breeding herd generation

The first goal of establishing an Oncopig breeding herd was to generate animals homozygous for the transgenes *TP53*^R167H^ and *KRAS*^G12D^. Prior to breeding, all animals were genotyped to confirm the presence of the transgenes (homozygous, heterozygous, or negative). Generation of the Oncopig breeding herd was accomplished by an initial cross of a Minnesota Minipig boar clone, heterozygous for the transgenes, with a Yorkshire dam that was negative for the transgenes. The resulting F1 offspring were confirmed to be either negative or heterozygous for the transgenes. Heterozygous F1 offspring were bred together to generate F2 offspring confirmed either homozygous, heterozygous, or negative for the TP53^R167H^ and KRAS^G12D^ transgenes. Only animals homozygous for the transgenes were kept as part of the breeding herd.

Our second goal was to generate Oncopigs homozygous for Swine Leukocyte Antigen (SLA). We were interested in a homozygous SLA because of the potential for future immunotherapeutic and cell/tissue donor/recipient grafting studies. To achieve this goal, Oncopigs homozygous for TP53^R167H^ and KRAS^G12D^ were haplotyped for SLA. Initial results showed a range of mixed class I and class II haplotypes, with complete SLA haplotypes including 3.3/35.12a, 3.3/35.13, 3.3/21.1, 2.19a/3.3, 3.3/68.19, and 3.3/3.3. Because 3.3 was the common haplotype for both classes, we kept and bred pigs with the goal of producing ones with the desired 3.3/3.3 complete haplotype. Once we produced enough boars and sows with the desired homozygous haplotype to keep our breeding herd going, those with heterozygous haplotypes were culled.

### 3.2. Oncopig size and growth rate

**Figure 1.**
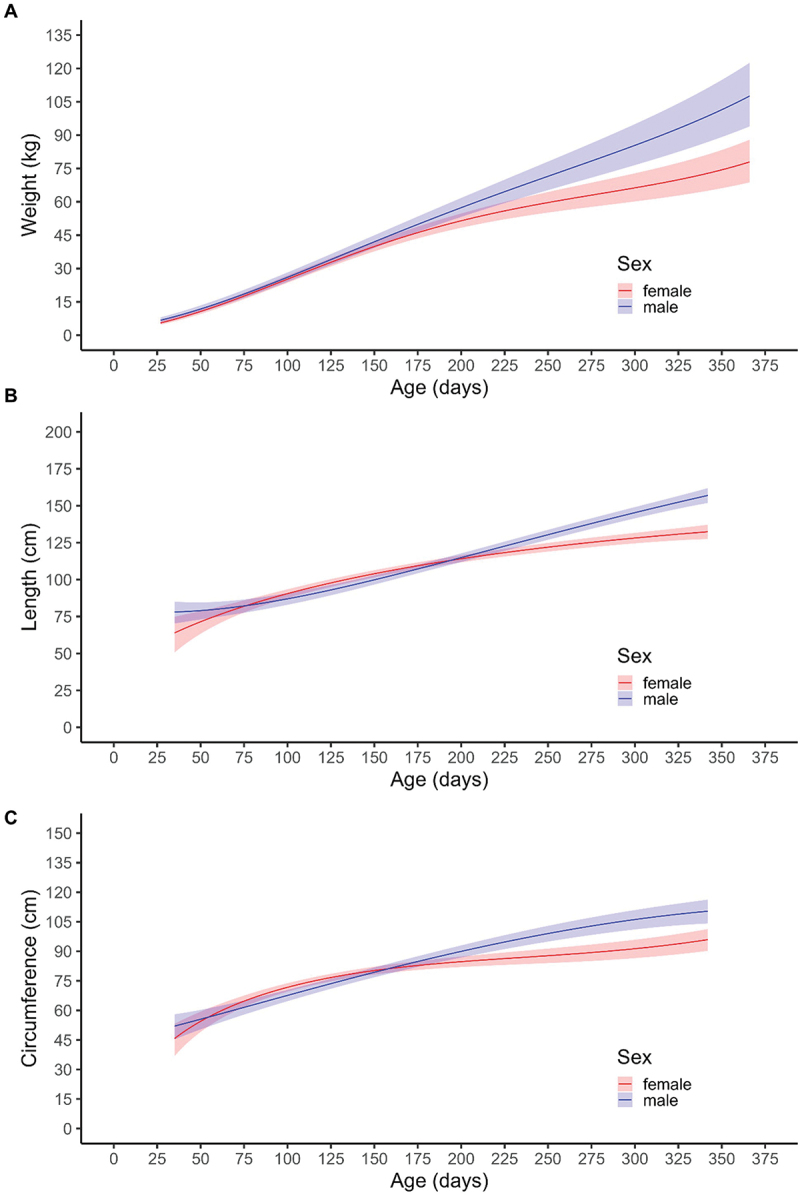
Mean body weight, length, and circumference of male and female Oncopigs from birth to one year of age based on linear mixed-effects models. Shaded areas represent 95% pointwise confidence bands around the mean. (A) Mean body weight (kg) of male (*n* = 4) and female (*n* = 6) Oncopigs from birth until nearly one year of age. (B) Mean body length (cm) of male (*n* = 4) and female (*n* = 6) Oncopigs from 1–12 months of age. (C) Mean body circumference (cm) of male (*n* = 4) and female (*n* = 6) Oncopigs from 1–12 months of age.

The body weight of male and female Oncopigs was similar between 0 and 6 months of age. Beyond 6 months of age, considered the age of sexual maturity in pigs, the male Oncopigs exhibited higher mean body weight compared to the female Oncopigs (Figure 1A). The mean body weight at 6 months of age was 52.3 kg (95% CI: 48.7, 56.1) for male Oncopigs and 48.0 kg (45.2, 50.8) for females. At 11 months of age, mean weight was 96.4 kg (85.2, 109) for male Oncopigs and 71.7 kg (64.1, 79.8) for female Oncopigs. In terms of growth rate, there was no statistically significant difference between male and female mean weight gain during the first 6 months. Between 0 and 6 months, the mean monthly growth rate was 8.97 kg (8.29, 9.66) for males and 8.34 kg (7.85, 8.85) for females, with an estimated rate difference of 0.628 kg per month (*p* = 0.987). Between 6 and 12 months, the monthly growth rate was 8.79 kg (6.72, 10.9) for males and 4.73 kg (3.27, 6.16) for females, with an estimated rate difference of 4.06 kg per month (*p* = 0.002), indicating a significantly higher growth rate for males over this period.

Male and female Oncopig body length and circumference were also determined from 1–2 months to 11 months old (Figure 1B,C). At 6 months of age, mean (95% CI) Oncopig length was 110 cm (107, 112) in males and 111 cm (109, 113) in females, while at 11 months of age, male and female Oncopig length was 155 cm (150, 160) and 132 cm (127, 136), respectively. Mean (95% CI) Oncopig circumference in male and female Oncopigs at 6 months of age was 86.5 cm (83.6, 89.3) and 83.5 cm (81.1, 85.8), respectively. At 11 months of age, circumference of male and female Oncopigs was 110 cm (104, 115) and 95.1 cm (89.5, 100), respectively. Both the length and circumference of Oncopigs showed no statistically significant differences between males and females between 1 and 6 months of age but were significantly greater in males at 6–11 months.

### 3.3. Analysis of Oncopig haematologic, coagulation, and serum biochemical parameters

Blood samples were collected from the ten Oncopigs at multiple timepoints between 1–12 months of age for analysis of 13 haematologic, 18 biochemical, and 3 coagulation parameters. Descriptive statistics for the haematologic parameters, including minimum, median, maximum, first quartile, and third quartile, are reported in Table 1. The 95% prediction intervals for the haematologic parameters were calculated for Oncopigs by age group (1–6 months or 6–12 months) and across the entire 1–12 months age range (Table 2 and Supplementary Table S6). These intervals are presented in Table 2 along with published reference intervals for domestic pigs and minipigs. The reference values for domestic pigs were established using arterial blood analysis from female Yorkshire swine (*Sus scrofa domesticus*) aged 3 to 4 months [11], while those for minipigs were based on data from female Yucatan minipigs aged 3 to 6 months, as reported in the Miniature Swine Book of Normals [16]. The 95% prediction intervals for red blood cells (RBC), haemoglobin, white blood cells (WBC), and platelets in Oncopigs were 6.48–9.08 ×10^6^/μL, 11.2–16.3 g/dL, 8.23–22.7 ×10^9^/L, and 198–556 ×10^9^/L, respectively.

**Table 1.** Descriptive statistics for haematologic parameters in Oncopigs.

Parameter	N	Minimum	Q1	Median	Q3	Maximum
Red blood cells (RBC) (x10^6^/μL)	168	5.74	7.33	7.99	8.52	9.52
Hemoglobin (g/dL)	168	9.80	13.3	14.6	15.4	16.9
Hematocrit (%)	167	36.2	42.1	44.9	48.2	58.2
Mean corpuscular volume (MCV) (fL)	166	47.7	53.7	57.0	59.0	69.0
Mean corpuscular haemoglobin (MCH) (pg)	159	16.1	17.6	18.0	18.8	20.9
Mean corpuscular haemoglobin concentration (MCHC) (g/dL)	164	27.6	31.2	32.3	33.3	35.4
Platelets (x10^9/L)	159	71.8	308	359	432	600
White blood cells (WBC) (x10^9^/L)	158	6.8	11.3	13.7	16.1	24.4
Absolute Segmented Neutrophils (x10^9^/L)	155	1.10	2.54	3.47	4.76	10.6
Absolute Lymphocytes (x10^9^/L)	152	3.34	6.84	8.69	11.1	20. 6
Absolute Monocytes (x10^9^/L)	156	0.092	0.312	0.554	0.749	2.31
Absolute Eosinophils (x10^9^/L)	131	0.052	0.191	0.300	0.438	0.920
Absolute Basophils (x10^9^/L)	99	0.008	0.032	0.052	0.112	0.530

N, number of observations; Q1, first quartile; Q3, third quartile.

**Table 2.** Haematologic parameters in Oncopigs, domestic pigs, and minipigs.

Parameter (units)	Oncopigs 95% prediction interval			Reference interval in domestic pigs	Reference interval in minipigs
	1–6 months old	+6–12 months old	combined (1–12 months old)		
Red blood cells (RBC) (x10^6^/μL)	6.56–8.81	6.42–9.19	6.48–9.08	4.80–7.11	6.18–8.35
Hemoglobin (g/dL)	11.0–15.5	11.7–16.5	11.2–16.3	8.20–11.7	10.8–15.3
Hematocrit (%)	36.6–49.8	36.5–54.3	36.4–53.1	25.4–38.8	31.6–48.2
Mean corpuscular volume (MCV) (fL)	49.8–64.2	51.6–66.5	50.3–65.8	N/A	50.2–61.7
Mean corpuscular haemoglobin (MCH) (pg)	16.8–18.7	17.1–20.7	16.9–20.5	N/A	16.5–19.6
Mean corpuscular haemoglobin concentration (MCHC) (g/dL)	27.6–34.9	29.9–34.6	28.2–34.8	N/A	30.7–35.0
Platelets (x10^9^/L)	244–573	178–511	198–556	162–449	229–633
White blood cells (WBC) (x10^9^/L)	10.7–23.9	7.60–18.4	8.23–22.7	9.9–22.0	10.5–26.0
Absolute Segmented Neutrophils (x10^9^/L)	1.67–9.43	1.42–6.98	1.50–8.56	1.96–8.81	1.1–15.5
Absolute Lymphocytes (x10^9^/L)	6.73–17.8	4.64–12.7	4.96–16.6	6.35–14.4	5.94–11.7
Absolute Monocytes (x10^9^/L)	0.209–2.79	0.134–1.40	0.152–2.32	0.06–0.41	0.33–1.46
Absolute Eosinophils (x10^9^/L)	0.085–0.848	0.095–0.924	0.089–0.903	N/A	0–0.75
Absolute Basophils (x10^9^/L)	0.023–0.941	0.013–0.210	0.015–0.690	N/A	0–0.15

The reference intervals for domestic pigs (female Yorkshire swine aged 3 to 4 months) and minipigs (female Yucatan minipigs aged 3 to 6 months) are extracted from previously published studies [11,16]. N/A, not available.

Eighteen serum biochemical parameters were analysed in Oncopigs in this study. Descriptive statistics for these parameters are presented in Table 3, and the 95% prediction intervals are reported for Oncopigs aged 1–6 months, 6–12 months, and the combined 1–12 month range in Table 4 and Supplementary Table S6, alongside published reference intervals for domestic pigs and minipigs [11,16]. The 95% prediction intervals for creatinine, albumin, and globulin in Oncopigs were 0.549–1.72 mg/dL, 3.24–4.44 g/dL, and 1.53–3.28 g/dL, respectively. For liver function tests, the 95% prediction intervals for Alkaline Phosphatase (ALP), Aspartate aminotransferase (AST), Gamma-glutamyl transferase (GGT) (U/L), and total bilirubin (mg/dL) were 71.7–280, 15.0–360, 34.1–69.1, and 0.076–0.444 respectively.

**Table 3.** Descriptive statistics for serum biochemical parameters in Oncopigs.

Parameter	N	Minimum	Q1	Median	Q3	Maximum
Creatinine (mg/dL)	173	0.600	1.10	1.30	1.50	1.90
Blood urea nitrogen (BUN) (mg/dL)	156	5.00	7.00	8.00	9.00	12.0
Total Protein (g/dL)	174	4.50	5.90	6.50	6.80	7.50
Albumin (g/dL)	168	3.10	3.60	3.70	3.90	4.70
Globulin (g/dL)	172	1.50	2.30	2.60	2.90	3.60
Calcium (mg/dL)	170	9.10	9.80	10.1	10.4	11.3
Phosphorus (mg/dL)	172	5.70	7.15	7.90	8.80	12.4
Sodium (mmol/L)	134	133	137	139	140	144
Potassium (mmol/L)	169	3.50	4.20	4.40	4.90	6.70
Chloride (mmol/L)	166	95.0	99.0	101	102	106
Magnesium (mg/dL)	164	1.90	2.20	2.30	2.50	2.90
Bicarbonate (mmol/L)	163	20.0	27.0	28.0	30.0	33.0
Alkaline Phosphatase (ALP) (U/L)	158	53.0	105	132	169	312
Aspartate aminotransferase (AST) (U/L)	168	15.0	22.0	28.5	45.5	225
Gamma-glutamyl transferase (GGT) (U/L)	170	30.0	40.0	47.0	53.0	98.0
Total Bilirubin (mg/dL)	174	0.100	0.100	0.200	0.200	0.900
Cholesterol (mg/dL)	169	53.0	69.0	78.0	88.0	139
Triglycerides (mg/dL)	169	13.0	22.0	29.0	36.0	89.0

N, number of observations; Q1, first quartile; Q3, third quartile.

**Table 4.** Serum biochemical parameters in Oncopigs, domestic pigs, and minipigs.

Parameter (units)	Oncopigs 95% prediction interval			Reference interval in domestic pigs	Reference interval in minipigs
	1–6 months old	+6–12 months old	combined (1–12 months old)		
Creatinine (mg/dL)	0.425–1.47	1.15–1.75	0.549–1.72	0.9–1.89	0.62–1.09
Blood urea nitrogen (BUN) (mg/dL)	4.97–10.2	6.13–14.6	5.26–13.8	2–10.9	6–17
Total Protein (g/dL)	4.70–6.83	6.02–7.25	4.88–7.17	5.1–7.19	5.7–7.6
Albumin (g/dL)	3.19–4.13	3.45–4.53	3.24–4.44	2.5–4.39	3.4–4.7
Globulin (g/dL)	1.35–3.0	1.98–3.35	1.53–3.28	3.9–4.2	1.8–3.5
Calcium (mg/dL)	9.45–11.0	9.38–10.8	9.39–10.9	9.2–11.2	9.2–11.3
Phosphorus (mg/dL)	7.22–10.9	6.14–9.75	6.29–10.6	6.8–10.6	6.6–10.2
Sodium (mmol/L)	133–143	134–143	133–143	129–143	131–147
Potassium (mmol/L)	3.88–6.15	3.71–5.68	3.77–6.0	3.5–4.7	3.5–8.6
Chloride (mmol/L)	96.9–104	97.0–105	96.9–104	N/A	93–108
Magnesium (mg/dL)	1.99–2.84	1.97–2.82	1.98–2.84	N/A	N/A
Bicarbonate (mmol/L)	18.8–31.6	24.3–32.4	20.1–32.2	N/A	18–26*
Alkaline Phosphatase (ALP) (U/L)	108–298	66.8–170	71.7–280	56–134	60–131
Aspartate aminotransferase (AST) (U/L)	17.0–467	14.2–230	15.0–360	N/A	26–312
Gamma-glutamyl transferase (GGT) (U/L)	37.4–70.7	33.1–66.5	34.1–69.1	N/A	37–62*
Total Bilirubin (mg/dL)	0.073–0.348	0.084–0.503	0.076–0.444	0.2–0.5	0.1–0.5
Cholesterol (mg/dL)	63.0–122	60.1–108	61.1–117	N/A	68–139
Triglycerides (mg/dL)	16.5–103	15.6–52.9	15.9–84.2	N/A	19–79

The reference intervals for domestic pigs (female Yorkshire swine aged 3 to 4 months) and minipigs (female Yucatan minipigs aged 3 to 6 months) are extracted from previously published studies [11,16]. N/A, not available; * data represents range rather than reference interval.

Descriptive statistics for coagulation parameters including prothrombin time, partial thromboplastin time, and fibrinogen are presented in Table 5, and the 95% prediction intervals for these parameters are reported in Table 6 and Supplementary Table S6. These coagulation parameters were reported for minipigs but not for domestic pigs in the cited studies [11,16]. For Oncopigs, the 95% prediction intervals for prothrombin time (PT), partial thromboplastin time (PTT), and fibrinogen were 10.7–18.3 seconds, 9.87–17.9 seconds, and 78.8–213 mg/dL, respectively.

**Table 5.** Descriptive statistics for coagulation parameters in Oncopigs.

Parameter	N	Minimum	Q1	Median	Q3	Maximum
Prothrombin Time (seconds)	122	10.0	12.3	13.7	15.1	19.8
Partial thromboplastin time (PTT) (seconds)	121	9.30	11.8	13.0	15.0	23.2
Fibrinogen (mg/dL)	89	60.0	118	143	163	220

N, number of observations; Q1, first quartile; Q3, third quartile.

**Table 6.** Coagulation parameters in Oncopigs and minipigs.

Parameter (units)	Oncopigs 95% prediction interval			Reference interval in minipigs
	1–6 months old	+6–12 months old	combined (1–12 months old)	
Prothrombin Time (seconds)	11.2–16.7	10.5–18.8	10.7–18.3	8.7–15.1
Partial thromboplastin time (PTT) (seconds)	9.81–17.5	10.0–18.1	9.87–17.9	14.5–51.8
Fibrinogen (mg/dL)	78.5–214	80.1–209	78.8–213	N/A

The reference intervals for minipigs (female Yucatan minipigs aged 3 to 6 months) are extracted from a previously published study [16]. N/A, not available.

## 4. Discussion

Pigs are valuable models for studying human diseases due to their physiological, anatomical, genetic, immunological, and drug metabolism similarities to humans [1–3]. The Oncopig Cancer Model, which harbours inducible TP53^R167H^ and KRAS^G12D^, allows for site-specific and temporally controlled tumour development [4,17]. Several cancer models, including liver, lung, pancreatic, and bladder cancers, have been developed using Oncopigs [18–26], with potential for more. This underscores the importance of having comprehensive data on Oncopig growth rates and serum biochemistry and haematology parameters.

In this study, 10 Oncopigs were monitored from birth to 12 months to evaluate growth rates, body size, and haematologic, biochemical, and coagulation parameters. As Oncopigs are a minipig/domestic pig hybrid, it is essential to determine their growth rates in comparison with both breeds. Our study demonstrates that Oncopigs have a body weight that falls between those of minipigs and domestic pigs. At six months of age, Oncopigs weigh approximately 50 kg, exceeding the body weight of Sinclair minipigs (19–23 kg) and Yucatan minipigs (26–30 kg) at the same age [16]. In contrast, male and female domestic pigs (Landrace × Large White) reach an average body weight of nearly 96 kg at five months of age [27]. The slower growth rate of Oncopigs relative to domestic pigs makes them more desirable and practical for biomedical research. Notably, male and female Oncopigs had similar body weights until six months of age, after which males exhibited higher body weights than females.

Furthermore, this study analyzes 13 haematologic, 18 biochemical, and 3 coagulation parameters, providing insights into the normal physiological phenotype of healthy Oncopigs that were not subjected to transgene expression. Although statistical comparisons were not conducted, the haematologic and biochemical parameters of normal Oncopigs appeared to be generally consistent with published reference intervals for domestic pigs and minipigs. In some instances, Oncopig values were more similar to one of the two breeds. For example, RBCs, haemoglobin, haematocrit, and platelet values were closer to those of minipigs, while the upper limit was higher than that in domestic pigs. In contrast, Oncopig WBCs, neutrophils, and lymphocytes were more similar to domestic pigs. For serum biochemical analysis, creatinine levels were similar to domestic pigs, while blood urea nitrogen and globulin levels were more similar to minipigs. Interestingly, the upper limit of ALP in Oncopigs was higher than those in both domestic pigs and minipigs. For coagulation parameters, PT upper limit was higher while PTT lower limit were lower than in minipigs. The availability of these haematologic, biochemical, and coagulation parameter values for Oncopigs is valuable for assessing the health status of tumour-bearing Oncopigs across various studies. While including control groups is necessary for research, the availability of physiological parameter values enables faster identification of abnormal results, saving time, resources, and animal lives.

A few limitations and considerations must be noted when interpreting the current data. A primary limitation is possible selection bias due to the small sample size of Oncopigs included in the study, which precluded the establishment of formal reference intervals [15,28]. Although the longitudinal study design yielded a substantial number of observations for each haematologic, coagulation, and serum biochemical parameter, the total number of animals assessed was limited to ten Oncopigs. According to the guidelines set by the American Society for Veterinary Clinical Pathology (ASVCP), a larger sample size is required for establishing de novo reference intervals [28]. Another limitation of the study is the small number of litters employed in the study; given that all female Oncopigs were from the same litter, and the male Oncopigs were from two litters, which might introduce genetic homogeneity and limit the data variability. Additionally, the study focuses on Oncopigs from birth to 12 months, which may not capture changes in haematologic and biochemical parameters beyond this age range. A further limitation of the study is the inconsistent timing of measurements across all animals included in the study. An important consideration when interpreting the data is that controlled environmental conditions and standardized diets have been used, which may not be identical in other research settings. Furthermore, the reported values are applicable to non-tumour-bearing Oncopigs that have not been subjected to transgene induction. Future studies should evaluate how transgene expression and tumour development affect these parameters.

## 5. Conclusions

In conclusion, this study describes the generation of Oncopig breeding herds and reports growth rate, body size, haematologic, coagulation, and serum biochemical parameters for normal non-tumour bearing Oncopigs. This study provides fundamental data critical for expanding the experimental use of Oncopigs for translational cancer research.

## Supplementary Material

Supplementary materials revision v2.docx

## Data Availability

All data generated during this study are included in this published article.
