# Supplementary material for: Analysis of growth rate, haematologic, and biochemical parameters of Oncopigs
Source: Int J Vet Sci Med. 2025 Jun 2;13(1):1–9. doi: 10.1080/23144599.2025.2502711 (PMC12131535; doi:10.1080/23144599.2025.2502711)
Supplement: Supplementary materials revision v2.docx [file TVSM_A_2502711_SM5648.docx]

**Supplementary Materials**

**Supplementary Table 1: Amount of feed provided to Oncopigs based on their weight**

| **Pig weight (kg)** | **Number of 8 oz. cups of feed** | **Amount of feed (kg)** |
| --- | --- | --- |
| 2 - 10 | 5 | 1.13 |
| 11 - 15 | 5.5 | 1.25 |
| 16 - 24 | 6 | 1.36 |
| 25 - 35 | 6.5 | 1.47 |
| 36 - 45 | 7 | 1.59 |
| 46 - 59 | 7.5 | 1.70 |
| 60 - 72 | 8 | 1.81 |
| 73 - 87 | 8.5 | 1.93 |

**Supplementary Table 2: Composition of diet provided to Oncopigs**

| **Grower diet 10900** | **%** |
| --- | --- |
| Corn, NRC, 2012 | 69.46 |
| Soybean meal, 48 %, NRC, 2012 | 27.21 |
| Choice white grease | 1 |
| Limestone | 0.96 |
| Dicalcium phosphate (DCP) | 0.92 |
| Swine^TM^ | 0.35 |
| Vit. ADEK | 0.1 |

NRC, National Research Council

**Supplementary Table 3: Age of Oncopigs (days) at weight measurement timepoints.**

|  | Female Oncopigs | | | | | | Male Oncopigs | | | |
| --- | --- | --- | --- | --- | --- | --- | --- | --- | --- | --- |
| Animal ID | 0299-16 | 0311-16 | 0319-16 | 0321-16 | 0327-16 | 0326-16 | 0624-17 | 0625-17 | 0627-17 | 0638-17 |
|  | 0 | 0 | 0 | 0 | 0 | 0 | 0 | 0 | 0 | 0 |
|  | 28 | 28 | 28 | 28 | 28 | 28 | 29 | 29 | 29 | 22 |
|  | 40 | 40 | 40 | 40 | 40 | 40 | 42 | 42 | 42 | 35 |
|  | 47 | 47 | 47 | 47 | 47 | 47 | 56 | 56 | 56 | 49 |
|  | 55 | 55 | 55 | 55 | 55 | 55 | 71 | 71 | 71 | 64 |
|  | 63 | 63 | 63 | 63 | 63 | 63 | 85 | 85 | 85 | 78 |
|  | 77 | 77 | 77 | 77 | 77 | 77 | 98 | 98 | 98 | 91 |
|  | 84 | 84 | 84 | 84 | 84 | 84 | 112 | 112 | 112 | 105 |
|  | 91 | 91 | 91 | 91 | 91 | 91 | 126 | 126 | 126 | 119 |
|  | 99 | 99 | 99 | 99 | 99 | 99 | 168 | 168 | 168 | 161 |
|  | 105 | 105 | 105 | 105 | 105 | 105 | 175 | 175 | 175 | 168 |
|  | 112 | 112 | 112 | 112 | 112 | 112 | 189 | 189 | 189 | 182 |
|  | 117 | 117 | 117 | 117 | 117 | 117 | 211 | 211 | 211 | 204 |
|  | 131 | 126 | 131 | 131 | 126 | 126 | 225 | 225 | 225 | 218 |
|  | 140 | 131 | 140 | 140 | 131 | 131 | 239 | 253 | 253 | 232 |
|  | 147 | 140 | 147 | 147 | 140 | 140 | 253 | 267 | 267 | 246 |
|  | 154 | 147 | 154 | 154 | 147 | 147 | 267 | 281 | 281 | 260 |
|  | 161 | 154 | 161 | 161 | 154 | 154 | 281 | 295 | 295 | 274 |
|  | 168 | 161 | 168 | 168 | 161 | 161 | 295 | 308 | 308 | 288 |
|  | 175 | 168 | 175 | 175 | 168 | 168 | 308 | 323 | 323 | 301 |
|  | 182 | 175 | 182 | 182 | 175 | 175 | 323 | 341 | 341 | 316 |
|  | 189 | 182 | 189 | 189 | 182 | 182 | 341 | 366 | 366 | 334 |
|  | 196 | 189 | 196 | 196 | 189 | 189 | 366 |  |  |  |
|  | 203 | 196 | 203 | 203 | 196 | 196 |  |  |  |  |
|  | 210 | 203 | 210 | 210 | 203 | 203 |  |  |  |  |
|  | 216 | 210 | 216 | 216 | 210 | 210 |  |  |  |  |
|  | 224 | 216 | 224 | 224 | 216 | 216 |  |  |  |  |
|  | 231 | 224 | 231 | 231 | 224 | 224 |  |  |  |  |
|  | 237 | 231 | 237 | 237 | 231 | 231 |  |  |  |  |
|  | 244 | 237 | 244 | 244 | 237 | 237 |  |  |  |  |
|  | 252 | 244 | 252 | 252 | 244 | 244 |  |  |  |  |
|  | 258 | 252 | 258 | 258 | 252 | 252 |  |  |  |  |
|  | 273 | 258 | 273 | 273 | 258 | 258 |  |  |  |  |
|  | 287 | 273 | 287 | 287 | 273 | 273 |  |  |  |  |
|  | 300 | 287 | 300 | 300 | 287 | 287 |  |  |  |  |
|  | 314 | 300 | 314 | 314 | 300 | 300 |  |  |  |  |
|  | 328 | 314 | 328 | 328 | 314 |  |  |  |  |  |
|  | 342 | 328 | 342 | 342 | 328 |  |  |  |  |  |
|  |  | 342 |  |  | 342 |  |  |  |  |  |
| Total number of measurements per Oncopig | 38 | 39 | 38 | 38 | 39 | 36 | 23 | 22 | 22 | 22 |

**Supplementary Table 4: Age of Oncopigs (days) at size measurement timepoints (length and circumference measurement).**

|  | Female Oncopigs | | | | | | Male Oncopigs | | | |
| --- | --- | --- | --- | --- | --- | --- | --- | --- | --- | --- |
| Animal ID | 0299-16 | 0311-16 | 0319-16 | 0321-16 | 0327-16 | 0326-16 | 0624-17 | 0625-17 | 0627-17 | 0638-17 |
|  | 55 | 55 | 55 | 55 | 55 | 55 | 42 | 42 | 42 | 35 |
|  | 77 | 77 | 77 | 77 | 77 | 77 | 56 | 56 | 56 | 49 |
|  | 105 | 105 | 91 | 91 | 105 | 105 | 71 | 71 | 71 | 64 |
|  | 117 | 117 | 105 | 105 | 117 | 117 | 112 | 98 | 112 | 91 |
|  | 131 | 131 | 117 | 117 | 131 | 131 | 126 | 112 | 126 | 105 |
|  | 147 | 147 | 131 | 131 | 147 | 147 | 140 | 126 | 140 | 119 |
|  | 161 | 161 | 147 | 147 | 161 | 161 | 154 | 140 | 154 | 133 |
|  | 175 | 175 | 161 | 161 | 175 | 175 | 175 | 154 | 175 | 147 |
|  | 189 | 189 | 175 | 175 | 189 | 189 | 189 | 175 | 189 | 168 |
|  | 203 | 203 | 189 | 189 | 203 | 203 | 211 | 189 | 211 | 182 |
|  | 216 | 216 | 203 | 203 | 216 | 216 | 225 | 211 | 225 | 204 |
|  | 231 | 231 | 216 | 216 | 231 | 231 | 239 | 225 | 239 | 218 |
|  | 244 | 244 | 231 | 231 | 244 | 244 | 253 | 239 | 253 | 232 |
|  | 258 | 258 | 244 | 244 | 258 | 258 | 267 | 253 | 267 | 246 |
|  | 273 | 273 | 258 | 258 | 273 | 273 | 281 | 267 | 281 | 260 |
|  | 287 | 287 | 273 | 273 | 287 | 287 | 295 | 281 | 295 | 274 |
|  | 300 | 300 | 287 | 287 | 300 | 300 | 308 | 295 | 308 | 288 |
|  | 314 | 314 | 300 | 300 | 314 | 314 | 323 | 308 | 323 | 301 |
|  | 328 | 328 | 314 | 314 | 328 | 328 | 341 | 323 | 341 | 316 |
|  | 342 | 342 | 328 | 328 | 342 | 342 |  | 341 |  | 334 |
|  |  |  | 342 | 342 |  |  |  |  |  |  |
| Total number of measurements per Oncopig | 20 | 20 | 21 | 21 | 20 | 20 | 19 | 20 | 19 | 20 |

**Supplementary Table 5: Age of Oncopigs (days) at blood draw timepoints.**

|  | Female Oncopigs | | | | | | Male Oncopigs | | | |
| --- | --- | --- | --- | --- | --- | --- | --- | --- | --- | --- |
| Animal ID | 0299-16 | 0311-16 | 0319-16 | 0321-16 | 0327-16 | 0326-16 | 0624-17 | 0625-17 | 0627-17 | 0638-17 |
|  | 56 | 56 | 55 | 55 | 56 | 56 | 56 | 42 | 56 | 35 |
|  | 105 | 105 | 78 | 77 | 105 | 105 | 71 | 56 | 71 | 49 |
|  | 117 | 117 | 92 | 92 | 117 | 117 | 85 | 85 | 85 | 64 |
|  | 131 | 131 | 105 | 105 | 131 | 131 | 98 | 98 | 98 | 78 |
|  | 147 | 147 | 117 | 117 | 147 | 147 | 112 | 112 | 112 | 91 |
|  | 161 | 161 | 131 | 131 | 161 | 161 | 126 | 126 | 126 | 105 |
|  | 175 | 175 | 147 | 147 | 175 | 175 | 140 | 140 | 140 | 119 |
|  | 189 | 189 | 161 | 161 | 189 | 189 | 155 | 155 | 155 | 133 |
|  | 203 | 203 | 175 | 175 | 203 | 203 | 175 | 175 | 175 | 148 |
|  | 216 | 216 | 189 | 189 | 216 | 216 | 189 | 189 | 189 | 168 |
|  | 231 | 231 | 203 | 203 | 231 | 231 | 211 | 211 | 211 | 182 |
|  | 244 | 244 | 216 | 216 | 244 | 244 | 225 | 225 | 225 | 204 |
|  | 258 | 258 | 231 | 231 | 258 | 264 | 253 | 253 | 253 | 218 |
|  | 287 | 273 | 244 | 244 | 273 | 273 | 271 | 282 | 271 | 236 |
|  | 314 | 287 | 258 | 258 | 287 | 287 | 282 | 295 | 295 | 246 |
|  | 328 | 300 | 273 | 273 | 314 | 300 | 295 | 341 | 323 | 264 |
|  | 342 | 314 | 287 | 287 | 328 |  | 323 | 366 | 341 | 275 |
|  | 363 | 328 | 314 | 300 | 342 |  | 341 |  | 366 | 288 |
|  |  | 342 | 328 | 314 | 363 |  | 350 |  |  | 318 |
|  |  |  | 342 | 328 |  |  | 366 |  |  | 334 |
|  |  |  | 363 | 342 |  |  |  |  |  |  |
|  |  |  |  | 363 |  |  |  |  |  |  |
| Number of blood samples collected per Oncopig | 18 | 19 | 21 | 22 | 19 | 16 | 20 | 17 | 18 | 20 |

**Supplementary Table 6: Oncopig hematology, biochemical, and coagulation parameters 95% prediction intervals with 90% confidence intervals (CI) around each endpoint.**

| Parameter (units) | **1 - 6 months old** | **+6 - 12 months old** | **Combined (1 - 12 months old)** |
| --- | --- | --- | --- |
| Absolute Basophils (x10^9/L) | 0.023 (0.017, 0.031) - 0.941 (0.554, 1.768) | 0.013 (0.010, 0.017) - 0.210 (0.151, 0.314) | 0.015 (0.012, 0.019) - 0.690 (0.417, 1.185) |
| Absolute Eosinophils (x10^9/L) | 0.085 (0.065, 0.110) - 0.848 (0.677, 1.098) | 0.095 (0.075, 0.119) - 0.924 (0.739, 1.163) | 0.089 (0.072, 0.108) - 0.903 (0.750, 1.092) |
| Absolute Lymphocytes (x10^9/L) | 6.733 (5.776, 7.607) - 17.809 (15.600, 20.466) | 4.642 (4.053, 5.290) - 12.657 (11.288, 14.625) | 4.964 (4.442, 5.624) - 16.578 (14.679, 18.914) |
| Absolute Monocytes (x10^9/L) | 0.209 (0.166, 0.264) - 2.787 (2.056, 4.050) | 0.134 (0.108, 0.166) - 1.401 (1.094, 1.787) | 0.152 (0.126, 0.182) - 2.315 (1.800, 3.146) |
| Absolute Segmented Neutrophils (x10^9/L) | 1.666 (1.379, 1.978) - 9.428 (7.723, 12.029) | 1.424 (1.177, 1.698) - 6.977 (5.937, 8.194) | 1.499 (1.277, 1.751) - 8.558 (7.320, 10.539) |
| Albumin (g/dL) | 3.189 (3.053, 3.310) - 4.131 (3.950, 4.358) | 3.452 (3.336, 3.579) - 4.528 (4.290, 4.811) | 3.235 (3.112, 3.354) - 4.442 (4.220, 4.698) |
| Alkaline Phosphatase (ALP) (U/L) | 107.934 (99.932, 116.067) - 297.754 (273.516, 326.568) | 66.785 (60.786, 72.731) - 169.523 (158.453, 181.935) | 71.652 (66.323, 77.134) - 279.735 (258.347, 303.895) |
| Aspartate aminotransferase (AST) (U/L) | 17.029 (15.281, 18.887) - 467.165 (145.760, --) | 14.218 (12.466, 15.881) - 229.847 (101.537, --) | 15.040 (13.552, 16.492) - 359.763 (143.503, --) |
| Bicarbonate (mmol/L) | 18.820 (15.971, 20.793) - 31.608 (30.876, 32.379) | 24.265 (23.367, 25.093) - 32.417 (31.680, 33.504) | 20.140 (17.740, 21.730) - 32.163 (31.565, 32.931) |
| Blood urea nitrogen (BUN) (mg/dL) | 4.974 (4.431, 5.483) - 10.211 (9.595, 10.905) | 6.134 (5.699, 6.591) - 14.628 (13.262, 16.144) | 5.262 (4.763, 5.718) - 13.783 (12.618, 15.105) |
| Calcium (mg/dL) | 9.445 (9.281, 9.604) - 10.959 (10.709, 11.257) | 9.379 (9.138, 9.562) - 10.821 (10.626, 11.041) | 9.394 (9.232, 9.556) - 10.906 (10.709, 11.139) |
| Chloride (mmol/L) | 96.942 (96.111, 97.778) - 104.401 (103.606, 105.288) | 97.042 (96.137, 97.849) - 104.513 (103.677, 105.312) | 96.930 (96.209, 97.646) - 104.493 (103.800, 105.255) |
| Cholesterol (mg/dL) | 63.034 (59.640, 66.748) - 121.521 (108.511, 140.090) | 60.118 (56.579, 63.578) - 107.764 (98.027, 121.524) | 61.129 (58.118, 64.282) - 116.585 (105.543, 132.042) |
| Creatinine (mg/dL) | 0.425 (-0.125, 0.589) - 1.470 (1.381, 1.556) | 1.146 (1.037, 1.248) - 1.746 (1.686, 1.807) | 0.549 (0.359, 0.671) - 1.716 (1.657, 1.773) |
| Fibrinogen (mg/dL) | 78.513 (62.038, 94.509) - 214.170 (197.365, 234.576) | 80.130 (66.223, 94.072) - 208.667 (191.339, 229.917) | 78.808 (65.592, 91.972) - 213.135 (199.021, 230.210) |
| Gamma-glutamyl transferase (GGT) (U/L) | 37.351 (34.574, 40.377) - 70.686 (61.196, 85.595) | 33.108 (30.996, 35.739) - 66.539 (57.735, 80.408) | 34.118 (32.069, 36.648) - 69.118 (60.099, 83.264) |
| Globulin (g/dL) | 1.353 (1.046, 1.600) - 3.001 (2.895, 3.119) | 1.976 (1.800, 2.151) - 3.351 (3.248, 3.457) | 1.525 (1.289, 1.719) - 3.277 (3.186, 3.369) |
| Hematocrit (%) | 36.559 (35.509, 37.721) - 49.784 (48.448, 51.334) | 36.517 (34.671, 38.133) - 54.315 (52.782, 56.019) | 36.442 (35.366, 37.422) - 53.123 (51.896, 54.476) |
| Hemoglobin (g/dL) | 10.995 (10.374, 11.526) - 15.521 (15.196, 15.863) | 11.732 (10.813, 12.447) - 16.541 (16.265, 16.842) | 11.168 (10.655, 11.666) - 16.328 (16.084, 16.586) |
| Magnesium (mg/dL) | 1.993 (1.942, 2.054) - 2.842 (2.716, 3.008) | 1.974 (1.877, 2.048) - 2.819 (2.716, 2.933) | 1.979 (1.919, 2.032) - 2.840 (2.746, 2.949) |
| Mean corpuscular hemoglobin (MCH) (pg) | 16.837 (16.485, 17.182) - 18.720 (18.297, 19.269) | 17.091 (16.759, 17.433) - 20.730 (20.235, 21.271) | 16.901 (16.564, 17.236) - 20.524 (20.036, 21.040) |
| Mean corpuscular hemoglobin concentration (MCHC) (g/dL) | 27.624 (26.700, 28.449) - 34.852 (34.405, 35.321) | 29.884 (29.322, 30.293) - 34.587 (34.166, 35.022) | 28.180 (27.395, 28.865) - 34.751 (34.406, 35.131) |
| Mean corpuscular volume (MCV) (fL) | 49.833 (48.694, 51.147) - 64.216 (61.511, 66.839) | 51.589 (50.261, 52.898) - 66.468 (63.872, 69.416) | 50.294 (49.192, 51.532) - 65.784 (63.382, 68.299) |
| Phosphorus (mg/dL) | 7.215 (6.989, 7.444) - 10.919 (10.319, 11.782) | 6.143 (5.960, 6.322) - 9.753 (9.290, 10.356) | 6.286 (6.123, 6.462) - 10.570 (10.082, 11.184) |
| Platelets (x10^9/L) | 244.431 (186.197, 294.486) - 573.191 (533.941, 620.235) | 177.859 (112.937, 232.898) - 510.858 (468.677, 556.607) | 197.917 (145.296, 246.352) - 555.613 (521.772, 596.811) |
| Potassium (mmol/L) | 3.881 (3.695, 4.072) - 6.153 (5.679, 6.841) | 3.714 (3.594, 3.840) - 5.680 (5.386, 6.058) | 3.768 (3.652, 3.878) - 5.995 (5.622, 6.529) |
| Prothrombin Time (seconds) | 11.186 (10.642, 11.690) - 16.656 (15.965, 17.550) | 10.467 (9.927, 10.976) - 18.815 (17.892, 19.882) | 10.705 (10.281, 11.123) - 18.282 (17.416, 19.161) |
| Partial thromboplastin time (PTT) (seconds) | 9.814 (9.237, 10.390) - 17.493 (16.117, 19.362) | 10.035 (9.492, 10.609) - 18.142 (16.788, 19.989) | 9.866 (9.399, 10.382) - 17.932 (16.744, 19.554) |
| Red blood cells (x10^6/μL) | 6.564 (6.336, 6.790) - 8.811 (8.502, 9.092) | 6.422 (6.033, 6.811) - 9.192 (8.868, 9.526) | 6.479 (6.189, 6.732) - 9.079 (8.756, 9.388) |
| Sodium (mmol/L) | 133.112 (130.715, 134.693) - 143.332 (142.363, 144.544) | 133.831 (132.838, 134.731) - 142.807 (141.903, 143.964) | 133.346 (131.628, 134.432) - 143.181 (142.411, 144.081) |
| Total Bilirubin (mg/dL) | 0.073 (0.063, 0.087) - 0.348 (0.263, 0.481) | 0.084 (0.071, 0.100) - 0.503 (0.374, 0.727) | 0.076 (0.066, 0.089) - 0.444 (0.344, 0.605) |
| Total Protein (g/dL) | 4.701 (4.354, 4.989) - 6.829 (6.664, 7.006) | 6.022 (5.815, 6.205) - 7.253 (7.099, 7.412) | 4.880 (4.593, 5.131) - 7.173 (7.025, 7.324) |
| Triglycerides (mg/dL) | 16.527 (14.383, 18.986) - 102.650 (68.820, 174.195) | 15.644 (13.861, 17.541) - 52.864 (44.103, 64.556) | 15.885 (14.221, 17.618) - 84.162 (61.471, 129.245) |
| White blood cells (WBC) (x10^9/L) | 10.728 (9.643, 11.921) - 23.943 (21.715, 26.626) | 7.604 (6.434, 8.651) - 18.419 (16.821, 20.337) | 8.234 (7.192, 9.227) - 22.664 (20.723, 24.963) |

The 95% prediction intervals are reported as Lower Bound (90% CI of Lower Bound) to Upper Bound (90% CI of Upper Bound).
